# Possible Crosstalk of the Immune Cells within the Lung and Mediastinal Fat-Associated Lymphoid Clusters in the Acute Inflammatory Lung Asthma-Like Mouse Model

**DOI:** 10.3390/ijms22136878

**Published:** 2021-06-26

**Authors:** Yaser Hosny Ali Elewa, Mahmoud Mansour Abd Elwakil, Osamu Ichii, Teppei Nakamura, Sherif Kh. A. Mohamed, Yasuhiro Kon

**Affiliations:** 1 Laboratory of Anatomy, Department of Basic Veterinary Sciences, Faculty of Veterinary Medicine, Hokkaido University, Hokkaido 060-0818, Japan; ichi-o@vetmed.hokudai.ac.jp (O.I.); nakamurate@vetmed.hokudai.ac.jp (T.N.); y-kon@vetmed.hokudai.ac.jp (Y.K.); 2 Department of Histology and Cytology, Faculty of Veterinary Medicine, Zagazig University, Zagazig 44511, Egypt; 3 Laboratory of Innovative Nanomedicine, Faculty of Pharmaceutical Sciences, Hokkaido University, Sapporo 060-0812, Japan; Mahmoud-nanomed@eis.hokudai.ac.jp; 4 Laboratory of Agrobiomedical Science, Faculty of Agriculture, Hokkaido University, Sapporo 060-0812, Japan; 5 Department of Biological Safety Research, Chitose Laboratory, Japan Food Research Laboratories, Hokkaido 066-0052, Japan; 6 Department of Anatomy and Embryology, Faculty of Veterinary Medicine, Zagazig University, Zagazig 44511, Egypt; SKMohamed@vet.zu.edu.eg

**Keywords:** asthma pathogenesis, lung injury, macrophages, mediastinal fat-associated lymphoid cluster, natural helper cells

## Abstract

Recently, we clarified the function of mediastinal fat-associated lymphoid clusters (MFALCs) in the progression of several respiratory diseases. However, their role has not yet been identified in the lung asthmatic condition. Hence, we compared the immune cells in lung and MFALCs of C57BL/6N mice on days 3 and 7 following intranasal instillation of either papain (papain group “PG”) or phosphate buffer saline (PBS) (vehicle group “VG”). The PG showed significantly prominent MFALCs, numerous goblet cells (GCs), and higher index ratios of different immune cells (macrophages, natural helper cells (NHC), B- and T-lymphocytes) within the MFALCs and lung than in the VG on both days 3 and 7. Interestingly, a tendency of decreased size of MFALCs and a significant reduction in the number of GCs and immune cells were observed within the MFALCs and lung in the PG on day 7 than on day 3. Furthermore, the quantitative parameters of these immune cells in MFALCs were significantly and positively correlated with the size of MFALCs and immune cells in the lung. This suggested that the possible crosstalk between immune cells within MFALCs and the lung could play a critical role in the progression and recovery of the acute inflammatory lung asthma.

## 1. Introduction

Asthma is a chronic inflammatory condition that affects the lower airways and is characterized by intermittent coughing, wheezing, and breathing difficulties [1,2]. A dramatic increase has been reported in the prevalence of asthma over the past 25 years [3], and up to 300 million asthma patients have been recorded worldwide [2]. Asthma could be induced by inhalation of various allergens (such as mold spores, pollen, mold, and dust mites) [1], air pollutants (such as smoke) [4], and protease allergen such as papain. The latter has been reported to cause occupational asthma in humans [5,6] and asthma-like symptoms in mice [7,8]. Asthma is one of the complex multifactorial diseases, thus several animal models were used to study their progression and pathogenesis following either acute or chronic exposure to irritants. Mouse models of the acute response have been used to identify the mechanisms and pathogenesis underlying the inflammatory and immunologic responses in asthmatic lung condition [9].

It has been clarified that infiltration of immune cells into the lung plays a critical role in the development of asthma followed by irritation in the airway [10,11]. Although the role of both T and B cells has been clarified in most mouse models that simulate asthma [12,13], recent studies have revealed the involvement of non-T and non-B innate lymphoid cells (ILCs) in the asthma development in the Rag-deficient mice lacking T and B cells following the administration of papain [14]. Macrophages and ILCs are tissue-resident innate immune cells that play important roles in tissue immunity and organ homeostasis [15]. Besides the role of macrophages in eliminating diseased and damaged cells, they are also considered as effector cells of the innate immune system because of their ability to present antigens to T cells [16,17]. Furthermore, the role of lung natural helper cells (NHC) as novel ILCs group2 (ILC2s) has been reported as a rapid T cell-independent source of Th2 cell-type cytokines population in papain-induced asthma mouse model [7,18]. The lung NHCs showed negative expression of mature hematopoietic lineage (Lin-) markers but were found to be Sca-1(+), c-Kit(+/lo), CD25(+), and CD127(+) cells [7].

Interestingly, a similar population of NHC was identified in novel lymphoid clusters associated with both the mesenteric and mediastinal fat tissue (MFT) in humans and mice. Such clusters are named as fat-associated lymphoid clusters (FALCs) [19,20]. Firstly, we discovered the FALCs associated with the MFT of healthy naïve mice and termed it mediastinal FALCs (MFALCs) and revealed their enrichment along with macrophages, B-lymphocytes, and T-lymphocytes, specifically T helper cells, and some NHC and granulocytes [20]. Notably, we reported more developed MFALCs in the inflammatory diseased condition of the lung, such as in the autoimmune disease mouse model and bleomycin-induced pneumonitis mouse model. Additionally, we clarified a significant positive correlation between the size of MFALCs and the degree of lung injury in such inflammatory conditions [21,22]. However, the role of MFALCs and the possible crosstalk between immune cells within both MFALCs and the lung is not yet clarified in the pathogenesis of the asthmatic condition.

In this study, we investigated the potential role of adaptive immune cells (B- and T-lymphocytes) and innate (macrophages and NHC) populations within the MFALCs in the progression and recovery of asthma in C57BL/6N (B6) mice following a single intranasal (i.n.) instillation of either papain (papain group) or PBS (vehicles group). We reported a significant increase in MFALCs’ size and the number of immune cells in bronchoalveolar lavage fluid (BALF), MFALCs, and lung lobes in papain group than that found in the vehicle group on both days 3 and 7. Furthermore, we revealed positive correlations between the immune cell infiltration in the lung along with both the size and immune cell populations of MFALCs. Such correlations showed significant values for B-lymphocytes, macrophages, and NHC but not for T-lymphocytes, suggesting possible crosstalk between the former immune cell populations within lung and MFALCs in either progression or recovery of the asthmatic condition. 

## 2. Results

### 2.1. Indices of Asthma Induction in the Mice Model

**Figure 1:**
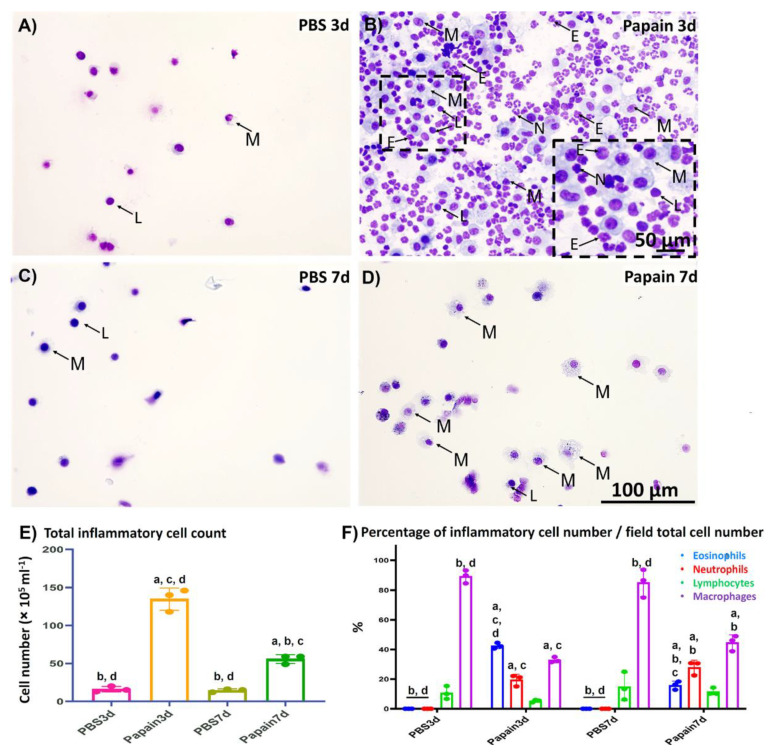
Cytological analysis of the bronchoalveolar lavage fluid (BALF) of papain groups and vehicle groups at 3 and 7d. (**A**–**D**) Diff-Quick-stained cytospin slides of vehicle group at 3d (**A**) and 7d (**C**), and papain group at 3d (**B**) and 7d (**D**). Numerous inflammatory cells (including lymphocytes “L”; macrophages “M”; eosinophils “E”, and neutrophils “N”) was observed in the papain group at 3d (**B**) than other groups. (**E**) Graph showing the total inflammatory cell count in the BALF of papain group and vehicle group at 3 and 7d. (**F**) Graph showing the differential leukocytic counts of the Diff-Quick-stained cytospin slides of BALF from papain group and vehicle group at indicated time points. The letters a, b, c, d: Significant differences between phosphate buffer saline (PBS) group at 3d (a), papain group at 3d (b), PBS group at 7d (c), and papain group at 7d (d), analyzed by the Kruskal–Wallis test, followed by the Scheffé’s method. (*p* < 0.05); n = 3/experimental group. Values are shown as the mean ± SE.

**Figure 2:**
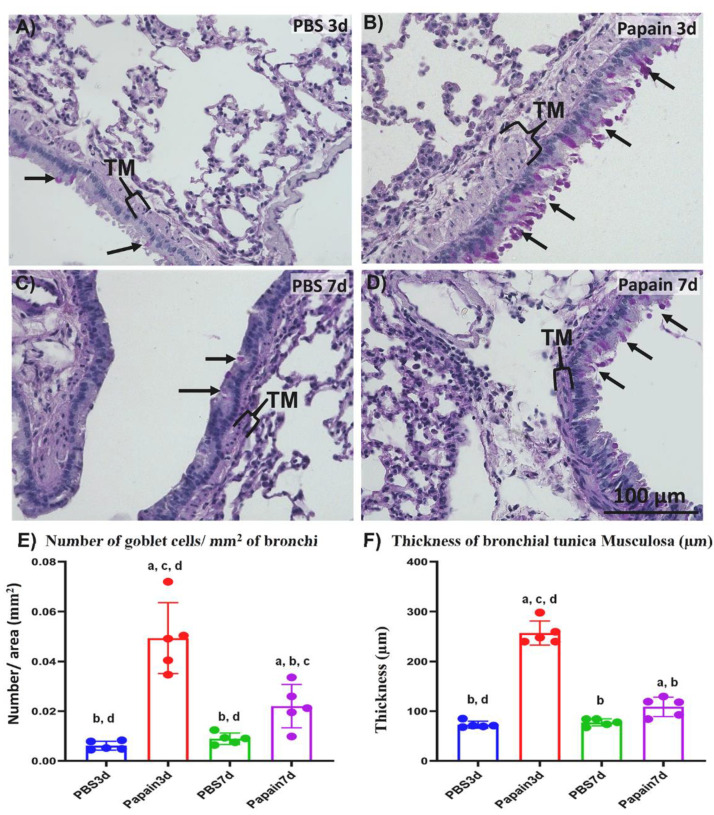
Histopathological features and morphometrical analysis of indices of lung asthma induction among papain groups and vehicle groups at 3 and 7d. (**A**–**D**) Histopathological features of PAS-Hx-stained lung sections of the vehicle group at 3d (**A**), and 7d (**C**) and papain group at 3d (**B**), and 7d (**D**). Notice goblet cells (arrows) and tunica musculosa of the bronchial wall (TM). (**E**) Graph showing the number of goblet cells in the bronchial lining epithelium for papain group and phosphate buffer saline (PBS) groups (vehicle group) at 3 and 7d. (**F**) Graph showing the average thickness of tunica musculosa of bronchial wall of lung tissue sections from papain group and vehicle group at indicated time points. The letters a, b, c, d: Significant differences between PBS group at 3d (a), papain group at 3d (b), PBS group at 7d (c), and papain group at 7d (d), analyzed by the Kruskal–Wallis test, followed by the Scheffé’s method. (*p* < 0.05); n = 5/experimental group. Values are shown as the mean ± SE.

To examine the degree of lung asthma induction among the studied groups, we compared the number of inflammatory cells within the BALF (Figure 1) along with both the number of goblet cells and the thickness of the tunica musculosa surrounding the bronchioles (Figure 2) between papain group and vehicle group on both days 3 and 7. 

#### 2.1.1. Analysis of Inflammatory Cells in the BALF

For analysis of the cytological changes in the BALF among various studied groups, we examined the Diff-Quick-stained cytospin sections (Figure 1A–D). Both the total number of inflammatory cells (Figure 1E) and their differential count (Figure 1F) were calculated and compared between papain group and vehicle group on days 3 and 7. 

As shown in Figure 1A–D, few inflammatory cells (mainly lymphocytes and macrophages) were observed in the Diff-Quick-stained cytospin sections in the vehicle group on days 3 and 7 (Figure 1A,C). On the other hand, a dramatic increase was detected in the inflammatory cells (mainly eosinophils, neutrophils, lymphocytes, and macrophages) in the sections of the papain group on day 3 (Figure 1B). Interestingly, examination of the cytospin sections from the BALF of papain group on day 7 revealed fewer immune cells (lymphocytes and macrophages) than those found on day 3, suggesting a recovery phase (Figure 1D). Similar to the cytological examination, the total number of inflammatory cells in the BALF was found to be significantly higher in the papain group compared to the vehicle group on both days 3 and 7. Furthermore, the papain group on day 7 showed a significant decrease in the total cell count than that found on day 3 (Figure 1E). For the differential cell count, we counted the number of various inflammatory cells (macrophages, lymphocytes, eosinophils, and neutrophils)/field and divided to the total cell count of the same field, and the percentages of various cells in the cytospined-stained sections were calculated and their averages were compared among the studied groups. For the percentage of macrophages, the vehicle group on days 3 and 7 showed significant higher percentages than that of the papain group. However, for the percentage of lymphocytes, no significant difference could be observed among the studied groups. On the other hand, the papain group showed a significantly higher percentage of both eosinophils, and neutrophils when compared to that of the vehicle group on days 3 and 7. Furthermore, a significant reduction of the percentage of eosinophils was observed in the papain group on day 7 than that of the papain group on day 3 (Figure 1F). 

#### 2.1.2. Morphological and Morphometrical Analysis of Lung Tissue among the Studied Groups

To examine the degree of lung asthma development, we compared the indices of asthma induction (the thickness of tunica musculosa around the bronchial wall and number of goblet cells in their lining epithelia) through the Periodic Acid Schiff-hematoxylin (PAS-Hx)-stained lung tissue sections of both vehicle group and papain group (Figure 2). Interestingly, histopathological examination of the lung tissue sections revealed thicker tunica musculosa and numerous goblet cells in the papain group compared to the vehicle group. Furthermore, more prominent tunica musculosa and goblet cells were observed in the papain group on day 3 when compared to those on day 7 (Figure 2A–D). 

Concomitant with the results of histopathological examination, a significantly higher value was observed in the number of goblet cells in the lung tissue of the papain group than that found in the vehicle group on both days 3 and 7. Moreover, a significant reduction was observed in such values in the papain group on day 7 compared to that on day 3 (Figure 2E). Similarly, on day 3, the papain group revealed a significant increase in the thickness of tunica musculosa than other studied groups as a characteristic lesion of lung asthma induction (Figure 2F). However, the papain group on day 7 showed a significant reduction in such values compared to that found on day 3. Additionally, the papain group on day 7 revealed significantly higher values than the vehicle group on day 3 and showed a tendency to have increased values compared to that of vehicle group on day 7 but without significant differences (Figure 2F). 

### 2.2. Morphological Features of MFALCs in the Papain Group vs. Vehicle Group

**Figure 3:**
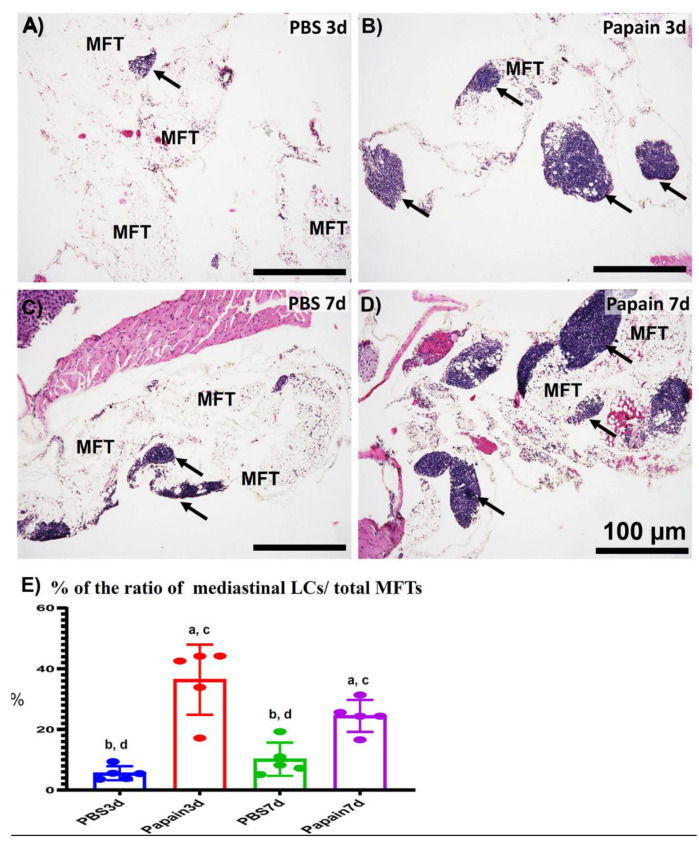
Histopathological features and morphometrical analysis of the degree of mediastinal fat-associated lymphoid clusters’ (MFALCs’) development among papain group and phosphate buffer saline (PBS) groups (vehicle group) at 3 and 7d. (**A**–**D**) Histopathological features of HE-stained mediastinal fat tissue (MFT) sections of the PBS groups at 3d (A) and 7d (**C**), and papain group at 3d (**B**) and 7d (**D**). Notice LCs (arrows) associating with the mediastinal fat tissue (MFT). (**E**) Graph showing the ratio of LCs/total MFT from papain group and vehicle group at indicated time points. The letters a, b, c, d: Significant differences between PBS group at 3d (a), papain group at 3d (b), PBS group at 7d (c), and papain group at 7d (d), analyzed by the Kruskal–Wallis test, followed by the Scheffé’s method. (*p* < 0.05); n = 5/experimental group. Values are shown as the mean ± SE.

To further investigate the effects of lung asthma induction on the degree of MFALCs through intranasal instillation of papain, we histologically compared the size of MFALCs between vehicle groups and papain groups. As shown in Figure 3, the papain group on both days 3 and 7 (Figure 3B,D) revealed prominent lymphoid clusters (LCs) compared to that of the vehicle group (Figure 3A,C). The area ratio of LCs/MFTs in H&E-stained sections was morphometrically measured to detect the histological index of MFALCs’ development among the studied groups. Our results revealed a significant increase in such ratios in the papain group than vehicle group on both days 3 and 7. Furthermore, the papain group showed decreased tendency in the values of such ratio on day 7 than that on day 3 without significant difference (Figure 3E). 

Similarly, the whole-mount hematoxylin-stained MFT revealed a more prominent cluster in the papain group than that in the vehicle group (Figure S1A–D). The morphometrical analysis indicated a significantly higher percentage of LCs area/total MFT area of the papain group when compared to that of the vehicle group. Additionally, a significant reduction in such percentage was observed in the papain group on day 7 than that on day 3 (Figure S1E).

### 2.3. Analysis of the Role of Peripheral Node Address in (PNAd) Positive High Endothelial Venules in the Development of MFALCs and Lung Asthma

**Figure 4:**
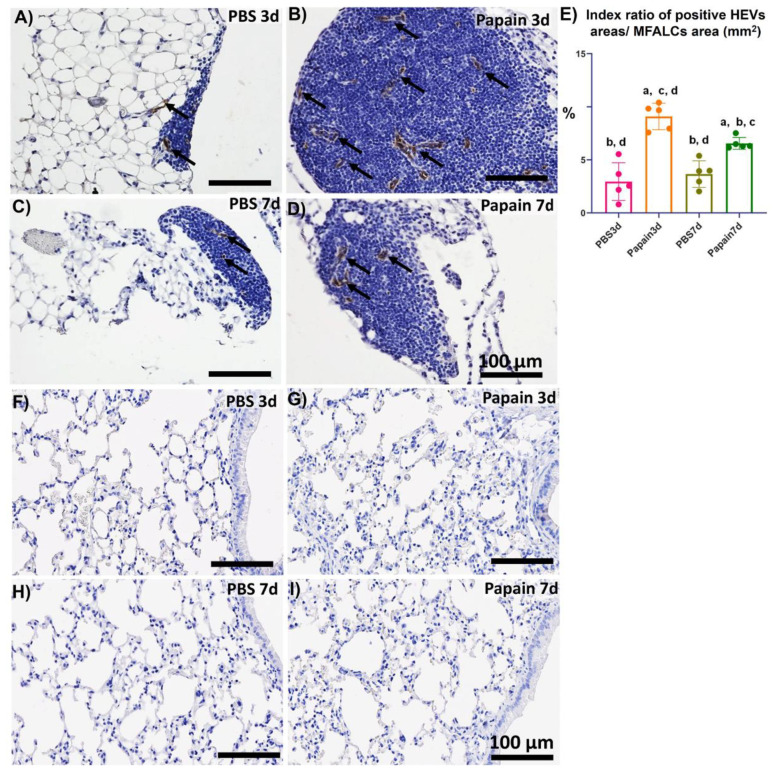
Analysis of the occurrence of PNAd^+^ high endothelial venules in the mediastinal fat-associated lymphoid clusters (MFALCs) and lung among papain group and phosphate buffer saline (PBS) groups (vehicle group) at 3 and 7d. (**A**–**D**) Histopathological features of immunohistochemical-stained mediastinal fat tissue sections of the PBS groups at 3d (**A**) and 7d (**C**), and papain group at 3d (**B**) and 7d (**D**). Notice PNAd^+^ high endothelial venules (arrows). (**E**) Graph showing the index ratio of PNAd^+^ HEVs’ areas/MFALCs’ area from papain group and vehicle group at indicated time points. The letters a, b, c, d: Significant differences between PBS group at 3d (a), papain group at 3d (b), PBS group at 7d (c), and papain group at 7d (d), analyzed by the Kruskal–Wallis test, followed by the Scheffé’s method. (*p* < 0.05); n = 5/experimental group. Values are shown as the mean ± SE. (**F**–**I**) Histopathological features of immunohistochemical-stained lung tissue sections of the PBS groups at 3d (**F**) and 7d (**G**), and papain group at 3d (**H**) and 7d (**I**). Notice the absence of PNAd^+^ high endothelial venules in the lung tissue sections of all studied groups.

Previously, we have clarified the role of high endothelial venules in the development of MFALCs and progression of inflammatory lung lesions in bleomycin-induced pneumonitis mice model [22]. Therefore, in the current investigation, we analyzed the occurrence of PNAd^+^ high endothelial venules by immunohistochemical-staining among different groups. Interestingly, more developed PNAd^+^ high endothelial venules were observed in the MFALCs of the papain group (Figure 4B,D) than that in the vehicle group at both time points (Figure 4A,C). Similarly, our morphometrical measurements of the index ratio of the PNAd^+^ high endothelial venules in MFALCs revealed their significant increase following papain administration especially on day 3 in comparison to that of the vehicle group. Additionally, a significant decrease in such ratio was observed in papain group on day 7 than that on day 3 (Figure 4E). On the other hand, no PNAd^+^ high endothelial venules were observed in the lung sections of both vehicle group and papain group (Figure 4F–I). These observations suggested that high endothelial venules could play a role in the development of MFALCs rather than inflammatory cells’ infiltrations into the lung tissue. 

### 2.4. Analysis of Immune Cells in MFALCs and Lung Tissues of Papain Group and Vehicle Group

**Figure 5:**
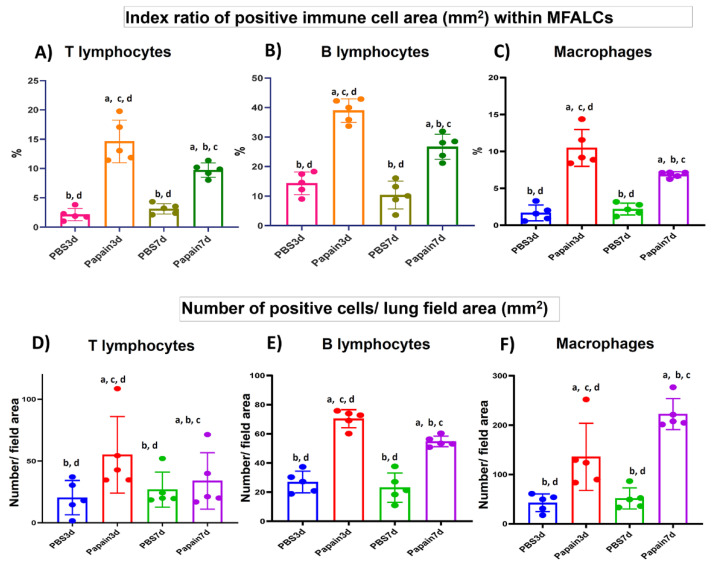
Analysis of the immune cell population in the mediastinal fat-associated lymphoid clusters (MFALCs) and lung of papain group and phosphate buffer saline (PBS) groups at 3 and 7d. (**A**-**C**) Graph showing the index ratio of positive immune cells area; CD3^+^ T-lymphocytes (**A**), B220^+^ B-lymphocytes (**B**), and Iba1^+^ macrophages (**C**) within MFALCs’ area of immunohistochemical-stained mediastinal fat tissue sections of the papain group and vehicle group at indicated time points. (**D**–**F**) Graph showing the index number of positive immune cells; CD3^+^ T- lymphocytes (**D**), B220^+^ B-lymphocytes (**E**), and Iba1^+^ macrophages (**F**) within the lung tissue sections of the papain group and vehicle group at indicated time points. The letters a, b, c, d: Significant differences between PBS group at 3d (a), papain group at 3d (b), PBS group at 7d (c), and papain group at 7d (d), analyzed by the Kruskal–Wallis test, followed by the Scheffé’s method (*p* < 0.05); n = 5/experimental group. Values are shown as the mean ± SE.

Immunohistochemical-staining was performed to detect the CD3^+^ T-lymphocytes (Figure S2), B220^+^ B-lymphocytes (Figure S3), and Iba1^+^ macrophages (Figure S4) within MFALCs and lung tissue in both papain group and vehicle group on day 3 and 7. Immunohistochemical analysis revealed a significantly higher index ratio of the positive area of the CD3^+^ T-lymphocytes, B220^+^ B-lymphocytes, and Iba1^+^ macrophages in the MFALCs of papain group than of the vehicle group on day 3. Moreover, a significant decrease in such ratio was observed in the papain group on day 7 than on day 3 (Figure 5A–C). Similarly, a significant increase was observed in the number of positive immune cells/lung area for CD3^+^ T-lymphocytes, B220^+^ B-lymphocytes, and Iba1^+^ macrophages in the papain group than in vehicle group in the lung tissue on both day 3 and 7 (Figure 5D–F). Further, the papain group on day 7 revealed a significant decrease in the number of CD3^+^ T-lymphocytes and B220^+^ B-lymphocytes/lung area than that observed on day 3. However, a significant increase was detected in the number of Iba1^+^ macrophages/lung area in the papain group on day 7 than that observed on day 3 (Figure 5F). 

### 2.5. Analysis of the Occurrence of NHC in MFALCs and Lung Tissues of Papain Group and Vehicle Group

**Figure 6:**
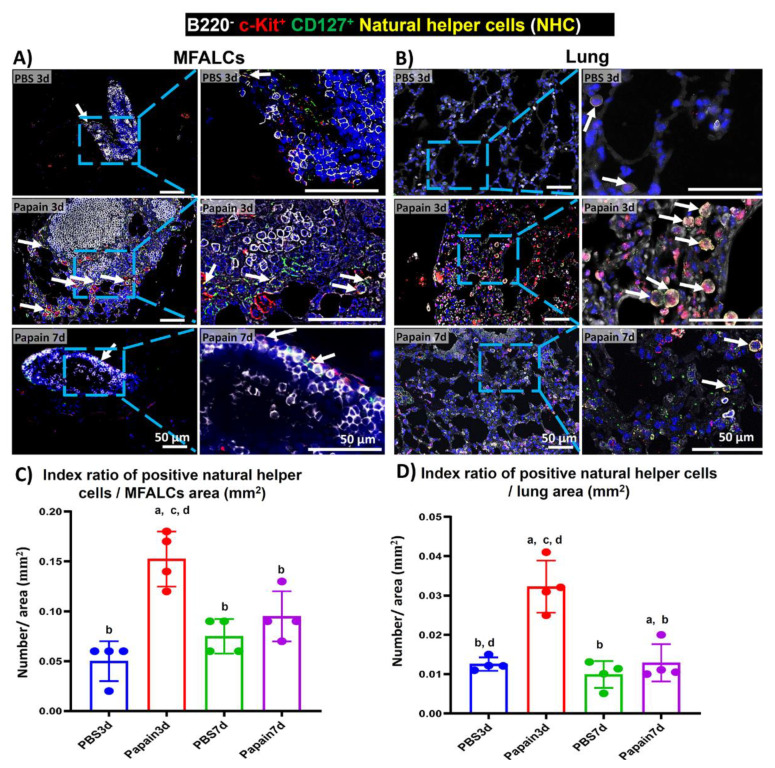
Analysis of the occurrence of natural helper cells (NHC) in the mediastinal fat-associated lymphoid clusters (MFALCs) and lung among papain group and phosphate buffer saline (PBS) groups at 3 and 7d. (**A**,**B**) Triple immunofluorescent-staining of the mediastinal fat tissue (MFT) (**A**) and lung sections (**B**) to detect B220 (white), c-Kit (red), and CD127 (green). Notice merged pictures showing B220^−^, c-Kit^+^, CD127^+^, NHC with yellow color (arrows), and B220^+^, c-Kit^−^, CD127^−^, B-lymphocytes (white-stained). (**C**) Graph showing the index ratio of NHC^+^ area/MFALCs’ field area of papain group and vehicle group at indicated time points. (**D**) Graph showing the index ratio of NHC^+^ area/lung field area of papain group and vehicle group at indicated time points. The letters a, b, c, d: Significant differences between PBS group at 3d (a), papain group at 3d (b), PBS group at 7d (c), and papain group at 7d (d), analyzed by the Kruskal–Wallis test, followed by the Scheffé’s method. (*p* < 0.05); n = 4/experimental group. Values are shown as the mean ± SE.

NHC is a new innate cell population reported in the LCs that is associated with the adipose tissue of the peritoneal [18] and mediastinal [19] cavities. The role of such cells was reported in the pathogenesis of lung asthma induction [7]. These cells were reported to express both c-Kit and CD127 but not lineage markers [18,19]. To detect the NHC in the MFALCs and lung tissues of different groups, we performed triple immunofluorescent staining against the B220 (lineage marker), c-Kit, and CD127. The staining revealed the existence of two main populations: B220^+^, c-Kit^–^, CD127^–^, B-lymphocytes, and B220^–^, c-Kit^+^, CD127^+^, NHC in both MFALCs and lung tissues of all studied groups (Figure 6A,B). Interestingly, the MFALCs and lung tissue in the papain group on day 3 showed numerous NHC than on day 7 and on both days 3 and 7 in vehicle group. The morphometrical measurement of the index ratio of the number of NHC/field area of either MFALCs or lung tissues revealed a significantly higher value in the papain group on day 3 compared to the vehicle group on both days 3 and 7. The papain group on day 7 showed a significant decrease in such ratio in both MFALCs and the lung in comparison to day 3. Additionally, there was a tendency to show higher values in papain group than that in the vehicle group on both days 3 and 7, which was significantly higher than that of the vehicle group on day 3 in lung tissue (Figure 6 C,D).

### 2.6. Histopathological Correlations between MFALCs’ Size, Indices of Lung Asthma Induction, High Endothelial Venules within MFALCs, and Immune Cells’ Infiltration in Lung

**Figure 7:**
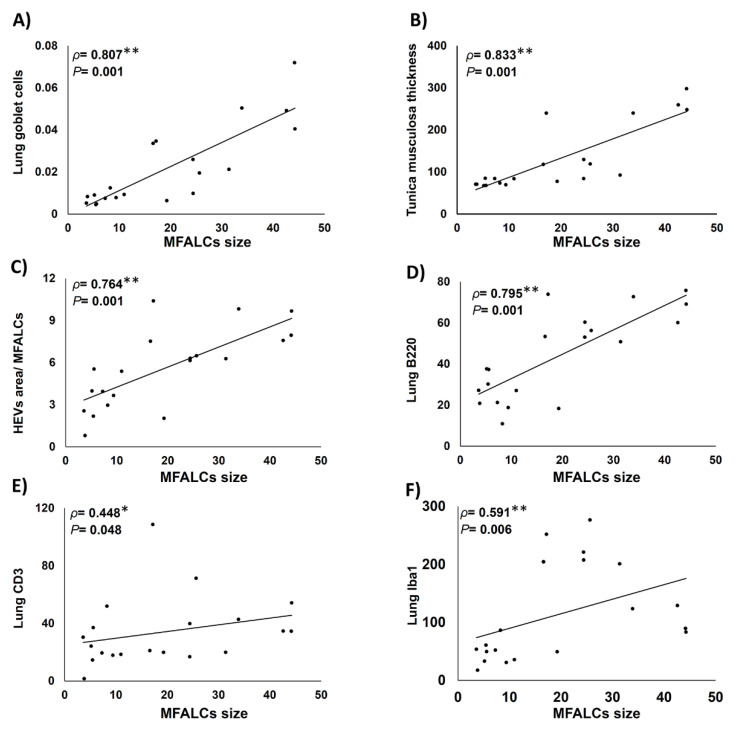
Analysis of the correlations between degree of development of mediastinal fat-associated lymphoid clusters (MFALCs) and the histopathological indices of lung asthma induction (number of goblet cells per bronchial wall, and the thickness of tunica musculosa of the bronchial wall), as well as the index ratio of high endothelial venules’ (HEVs) area/MFALCs’ area, and the parameters of immune cell infiltrating lung tissue (including the CD3^+^ T-lymphocytes, B220^+^ B-lymphocytes, and Iba1^+^ macrophages). (**A**–**E**) Graph showing Spearman’s correlations between MFALCs’ size and the number of GC per bronchial wall (**A**); thickness of tunica musculosa of the bronchial wall (**B**); index ratio of HEVs’ area/MFALCs’ field area (**C**); CD3^+^ T-lymphocytes (**D**); B220^+^ B-lymphocytes (**E**), and Iba1^+^ macrophages (**F**) in the infiltrating lung tissue. Data was analyzed by the Spearman’s correlation test, ρ: Spearman’s rank order correlation coefficient, n = 20, *: Significant, *p* < 0.05. **: Highly significant, *p* < 0.01.

As shown in Figure 7, we examined the Spearman’s correlations between the size of MFALCs and the histopathological indices of lung asthma induction (number of GC per bronchial wall and the thickness of tunica musculosa of the bronchial wall) along with the index ratio of high endothelial venules’ area/MFALCs’ area, and the parameters of immune cell infiltrating lung tissue (including the CD3^+^ T-lymphocytes, B220^+^ B-lymphocytes, and Iba1^+^ macrophages). Highly significant positive correlations were observed between the MFALCs’ size and the histopathological parameters of lung asthma induction (Figure 7A,B) along with that of the degree of high endothelial venules development (Figure 7C). Furthermore, our data analysis revealed positive correlations between MFALCs’ size and immune cell infiltration in the lung. Such correlations showed highly significant values for the lung B220^+^ B-lymphocytes (Figure 7D) and, Iba1^+^ macrophages (Figure 7F), and significant difference was reported for the lung CD3^+^ T-lymphocytes (Figure 7E).

### 2.7. Histopathological Correlations between Immune Cell Populations in MFALCs and Infiltration in the Lung 

**Figure 8:**
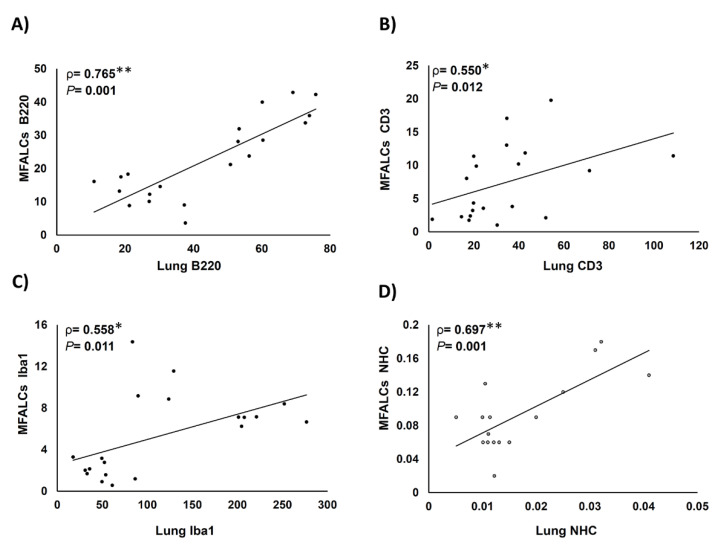
Analysis of the correlations between immune cell population within mediastinal fat-associated lymphoid clusters (MFALCs) and within the lung tissue. (**A**–**D**) Graph showing Spearman’s correlations between immune cells’ populations in MFALCs and in the lung tissue. Correlations between MFALCs and lung B220^+^ B-lymphocytes (**A**); CD3^+^ T-lymphocytes (**B**); Iba1^+^ macrophages (**C**), and natural helper cells (NHC) (**D**)**.** Analyzed by the Spearman’s correlation test, ρ: Spearman’s rank order correlation coefficient, n = 20 for B220^+^ B-lymphocytes, CD3^+^ T-lymphocytes, and Iba1^+^ macrophages, and n = 16 for NHC. *: Significant, *p* < 0.05. **: Highly significant, *p* < 0.01.

We analyzed the correlations between the index ratio of the positive area of immune cells (B220^+^ B-lymphocytes, CD3^+^ T-lymphocytes, Iba1^+^ macrophages, and NHC) in MFALCs and the number of similar immune cell infiltration in the lung tissue sections (Figure 8). We revealed positive correlations between the immune cell population in MFALCs with that of the population in lung tissue. Highly significant positive correlations were observed in the parameters of B220^+^ B-lymphocytes (Figure 8A), and NHC (Figure 8D). However, for the correlation of the CD3^+^ T-cell populations, and Iba1^+^ macrophages between MFALCs and the lung, significant values were observed (Figure 8B,C). 

## 3. Discussion

Asthma is one of the chronic lung diseases affecting large groups of people worldwide [23]. Currently, there is no curable medication for the asthmatic condition, except for continuous symptomatic therapy. Therefore, understanding the pathogenesis of asthma progression could provide innovative strategies for its treatment. Due to ethical reasons in humans, animal models, particularly mice, were frequently used for investigating the pathogenesis and mechanisms of asthmatic progression [9]. It was reported that the mouse models of allergic asthma could be induced either by acute (single exposure) or chronic (repeated exposure) allergen challenge [9]. For investigating the structural changes associated with asthma progression, several studies used the chronic challenged mouse models [24,25]. However, to elucidate the mechanisms underlying the immunological and inflammatory responses in asthma, only a few reports used acute asthmatic mouse models following a single exposure to papain. Furthermore, no reports were presented concerning the possible crosstalk between immune cells within MFALCs and lung in the pathogenesis of asthmatic condition. Therefore, we characterized the cellular composition of the clusters and lung infiltrations following a single dose administration of either papain or PBS at two time points (3 and 7d). Additionally, we analyzed the correlation between MFALCs’ development and the progression or recovery of lung asthma.

Human allergic asthma is characterized by airway inflammation, which is followed by airway remodeling and structural changes, including smooth muscle thickening, goblet cells hyperplasia/metaplasia, and increased vascularity [26,27]. Similarly, our results revealed significantly higher asthma indices (goblet cells’ number and thickness of tunica musculosa within the bronchial wall), as well as the total inflammatory cell counts within BALF of papain group compared to the vehicle group on both days 3 and 7, indicating that a single instillation of papain mimics pathogenesis of asthmatic conditions. Interestingly, a significant reduction in the former parameters was observed in the papain group on day 7 than on day 3, suggesting a recovery phase (on day 7) after the asthma progression (on day 3).

Previous reports revealed that inflammation could induce the formation and development of FALCs in the peritoneal and mediastinal fat tissue [22,28]. Therefore, we assessed the effect of asthma induction by papain administration on days 3 and 7 on the development of MFALCs. The papain group showed more developed MFALCs than the vehicle group. Furthermore, a significant reduction was observed in the MFALCs’ size in the papain group on day 7 than on day 3. These results indicated that the development of MFALCs could be induced by the presence of irritants in the lung, and the size of clusters could be decreased with the disappearance of the effect of such irritants. Previously, we clarified the role of high endothelial venules in the development of MFALCs in the bleomycin (BLM)-induced pneumonitis mouse model [22]. Similarly, the current investigation revealed a significant positive correlation between the size of MFALCs and high endothelial venules. Further, we had previously revealed the occurrence of HEVs in the lung of the BLM-induced pneumonitis group but not in the PBS control group. This suggests their role in the development of lung injury through both recruitment of inflammatory cells into MFALCs and migration into lung tissue in the inflammatory lung condition following the administration of BLM [22]. On the other hand, no high endothelial venules were observed in the lung of papain group and vehicle group but were well developed in MFALCs of papain group, suggesting a lesser role of high endothelial venules in the migration of immune cells into the lung but an obvious role in the development of MFALCs via recruitment of immune cells into the LCs in case of lung asthma condition.

Interestingly, previous studies revealed the cellular composition of such FALCs, including T- and B-lymphocytes and macrophages in addition to unique innate lymphoid cell populations termed NHC [19,20]. Despite the presence of several reports concerning the role of several immune cells (T- and B-lymphocytes and macrophages) [10,11,12,13] and the lung NHC [7] in the pathogenesis of lung asthma progression, the possible source of the recruitment of immune cells in the asthmatic mouse model is still poorly understood. Currently, we revealed the correlation between the development of MFALCs and pathogenesis of several lung injuries and immune cell infiltration into the lung using several mouse models, including genetic autoimmune mouse model [21,29] with the septic condition such as bleomycin-induced pneumonitis mouse model [22], the aseptic condition such as *Mycoplasma pulmonis* infection in mice [30], and metabolic disorders such as streptozotocin-induced diabetic mouse model [31]. However, the functional attribution of MFALCs in either asthma progression or recovery is not yet clarified. 

We have reported a significant increase in the immune cell populations in both MFALCs and lung tissue in papain group than in the vehicle group, and also revealed that the papain group on day 7 showed a significant reduction in such populations in comparison to day 3, suggesting a recovery phase. Furthermore, we have clarified a significant positive correlation between the number of immune cells infiltrating the lung tissue and the size of MFALCs. Therefore, it is conceivable that MFALCs’ development could contribute to lung asthma pathogenesis and might describe the possible crosstalk between immune cell populations within MFALCs and the infiltration in the lung tissue especially in the acute inflammatory lung asthma-like lesions. Interestingly, among the immune cell population within MFALCs and lung, significant positive correlations were observed between the B-lymphocytes, T-lymphocytes, macrophages, and NHC. Therefore, our results strongly suggest the possible crosstalk of such immune cell populations within MFALCs and lung tissue. Moreover, our data might reflect a significant possible role of MFALCs in the pathophysiology of lung asthma progression and regression. However, further investigations are necessary to confirm the recruitment of various immune cells within the MFALCs and lung tissue, as well as to examine the effect of papain inhalation as single dose (acute asthma-like lesions) as well as following sensitization (chronic stage) on T cell-deficient strain and B cell-deficient strain and compared with other asthma mouse models. Moreover, there is a need to analyze different micro-environmental factors controlling such possible recruitment. Understanding such factors could provide innovative therapeutic strategies for acute inflammatory lung asthma and other respiratory diseases via targeting and reprogramming of immune cells within the MFALCs.

## 4. Materials and Methods

### 4.1. Mice

Thirty-two male B6 mice (aged 12 weeks) were used in the present study. Mice were purchased from Japan SLC, Inc. (Shizuoka, Japan) and maintained under specific pathogen-free conditions in standard laboratory cages with access to water and rodent chow *ad libitum*. This study was approved by the Institutional Animal Care and Use Committee of the Graduate School of Veterinary Medicine, Hokkaido University (approval number: 15–0079). All the used animals were approved by the Association for Assessment and Accreditation of Laboratory Animal Care (AAALAC) International as per the Guidelines for the Care and Use of Laboratory Animals, Graduate School of Veterinary Medicine, Hokkaido University.

### 4.2. Experimental Design

B6 mice were slightly anesthetized with an intraperitoneal (i.p.) injection of 0.1 mL/10 gm body weight (B.W.) using anesthetic solution (mixture of 5.0 mg/kg B.W. butorphanol, 4.0 mg/kg B.W. midazolam, and 0.3 mg/kg B.W. medetomidine). These mice were divided into two cohorts: the papain group and vehicle group, and each cohort consisted of 16 mice. Following the anesthesia, the mice were administered 50 µL of single i.n. instillations of either PBS (vehicle group) or papain (from Carica papaya, Roche Diagnostics GmbH, Mannheim, Germany, lot number: 11819824, catalog number: 10108014001). For papain group, we used a dose of 10 mg/kg of B.W., which was based on the results of the histopathological examination of BALF and lung tissue for our preliminary experiments using various concentrations of papain (5, 10, 15, 20 mg/kg B.W.) (Figure S5). After papain or PBS instillation on days 3 (early time point) and 7 (late time point), the mice were euthanized using the previous anesthetic solution at a dose of 0.2 mL/10 gm; following which, the BALF, lung tissue, and MFTs were immediately collected for further analysis.

### 4.3. BALF Collection and Preparation for Cytological Interpretation

For the analysis of the cellular content within the BALF among different groups, we used 6 mice/group (3 mice/time point) and collected the fluid according to our previous method [29]. The collected BALF from different mice groups was subjected to centrifugation at 300× *g* for 5 min at 4 °C to obtain the cell pellets, which were then resuspended in 1.5 mL of distilled water (DW). This was kept for 10 s and then 500 µL of 0.6 M KCl was added to the suspension and mixed thoroughly. The suspensions were centrifuged again at the same speed and the obtained cell pellets were resuspended in 500 µL of sterile saline (0.9% NaCl). The total numbers of BALF cells were counted using a disposable hemocytometer (Funkoshi Co., Ltd., Tokyo, Japan, code number: 521–10).

For determining the differential BALF cell counts, 200 µL of the suspension was placed onto a Shandon™ EZ Single funnel (Thermo Fisher Scientific, Cheshire, UK) and centrifuged at 108× *g* using a cytocentrifuge for 10 min. The cytospin sections were stained with a Diff-Quick Staining Kit (Sysmex, Hyogo, Japan) according to the manufacturer’s protocol. The number of eosinophils, neutrophils, lymphocytes, and macrophages/field was scored using digital images from slides captured by the BZ-X710 microscope (Keyence, Osaka, Japan) and the averages for the percentage of different cells/field total cell count were calculated and compared among studied groups.

### 4.4. Morphological and Histopathological Analysis

For both morphological and histopathological analysis, mice were perfused with cold PBS from the left ventricle. Both MFTs and the right lung lobes were harvested from 20 mice (five mice/group at the indicated time points) and fixed immediately in 4% paraformaldehyde overnight. For morphological examination of the MFALCs, the whole-mount hematoxylin method was performed for the MFT followed by overnight fixation. The stained MFT was observed under a stereomicroscope after penetration by glycerol, according to our previous study [20]. For the histopathological analysis, the MFT and lung specimens were washed, dehydrated through a series of ascending grades of alcohol, cleared in pure xylene, and finally embedded in paraffin. Paraffin sections of 3 µm thickness were prepared from both MFTs and lung for histopathological analysis. The deparaffinized sections were then subjected to different staining methods namely hematoxylin and eosin (H&E) staining, periodic acid Schiff-hematoxylin (PAS-Hx), and immunohistochemical and immunofluorescent staining.

### 4.5. Immunohistochemical Staining of High Endothelial Venules and Immune Cells

To examine the occurrence of high endothelial venules within the MFALCs and lung tissues, immunohistochemical staining was performed using rat anti-PNAd antibody (BioLegend, San Diego, CA, USA). Furthermore, to detect different immune cells (T-lymphocytes, B-lymphocytes, and macrophages), immunohistochemical staining was performed using rabbit monoclonal anti-CD3, rat monoclonal anti-B220, and rabbit polyclonal anti-Iba1 antibodies, respectively, according to our previous report [29]. The deparaffinized sections were rehydrated and subjected to the heat-mediated antigen retrieval with either 10 mM Tris-HCl buffer (pH 7.4) (for PNAd and CD3) or 10 mM citrate buffer (pH 6.0) (for B220 and Iba1) at 105 °C for 20 min. After cooling, the sections were incubated in 0.3% H_2_O_2_ in methanol at room temperature for 20 min, followed by washing in DW. All sections were then incubated with 10% normal goat serum in a humid chamber for 1 h at room temperature. Next, the sections were incubated overnight at 4 °C with specific primary antibodies diluted in PBS (1:500 for PNAd, 1:200 for CD3, 1:1600 for B220, and 1:1200 for Iba1 immunostaining). These sections were washed three times in PBS for 5 min each, followed by 30 min incubation with specific biotinylated secondary antibodies (goat anti-rat IgM for PNAd, goat anti-rat IgG for B220 and goat anti-rabbit IgG for CD3 and Iba1) at room temperature. The sections were then washed with PBS, followed by incubation with streptavidin for 30 min at room temperature, which was subjected to a three-time wash with PBS (5 min/each). Finally, the positive reactions were visualized, followed by short incubation (1–2 min) of the sections with 3, 3′ diaminobenzidine tetrahydrochloride-H_2_O_2_ solution, where the nuclei were lightly stained with hematoxylin. The sections were then dehydrated in ascending grades of alcohol, cleared in xylene, mounted in non-polar mounting media, and examined under a light microscope.

### 4.6. Immunofluorescent Staining of NHC

To detect NHC, we performed a triple immunofluorescence staining for B220 as a Lin marker for B-lymphocytes, c-Kit, and CD127 in both MFTs and lung tissues according to our previous report [20], and the results were compared among the groups. NHCs were reported to be c-Kit^+^ and CD127^+^ but were negatively stained with the Lin markers [7,19]. Briefly, after deparaffinization and rehydration, the sections were treated with 10 mM citrate buffer (pH 6.0) for 20 min at 105 °C, followed by incubation with normal donkey serum at room temperature for one hour in a humid chamber. Then, the sections were incubated overnight with a mixture of rat anti-B220 antibody (1:1600), goat anti-c-Kit antibody (1:100), and rabbit anti-CD127 antibody (1:1000). Next, the sections were incubated for 30 min with a mixture of Alexa-Fluor-488-labeled donkey anti-rat IgG, Alexa-Fluor-546-labeled donkey anti-goat IgG, and Alexa-Fluor-647-labeled donkey anti-rabbit IgG (Invitrogen, Eugene, Oregon, USA) at a dilution of 1:500, followed by Hoechst 33342 (1:2000) (Dojindo, Kumamoto, Japan) incubation for 3 min. The sections were washed with PBS (3 times/5 min each) and mounted using Fluoromount (catalog number: K024, Diagnostic BioSystems, USA). Immunofluorescent signals were examined using the BZ-X710 (Keyence, Osaka, Japan).

### 4.7. Histomorphometric Measurements

For the histomorphometric analysis, the H&E-, PAS-Hx- and immuno-stained sections of MFTs and lung were analyzed between papain group and vehicle group on both days 3 and 7 (n = 5 in each condition). To compare the MFALCs’ size among the groups, digital images from H&E-stained sections were captured by BZ-X710 microscope. Both the LC area and total MFT area were measured using the ImageJ software (ver. 1.32j, http://rsb.info.nih.gov/ij (accessed on 18 May 2021)), and then, the ratio of LC area/total MFT area was reported as recorded previously [20]. To analyze the changes in goblet cells as a parameter of asthma development, the PAS-Hx-stained sections were converted into virtual slides by NanoZoomer 2.0-RS (Hamamatsu Photonics K.K., Japan), and then the number of GC/mm^2^ of bronchi was analyzed using NDP.view2 program (Hamamatsu Photonics K.K.). For immuno-stained sections, the digital images were captured from MFTs and lung sections using the BZ-X710 microscope to analyze the ratios of positive areas for macrophages, B-, and T-lymphocytes’/MFALCs’ area (mm^2^), number of positive cells (NHC, macrophage, B-, and T-lymphocytes)/lung lobe area (mm^2^), and the number of positive NHC/MFALCs’ area (mm^2^) using the BZ-X analyzer (Keyence). 

### 4.8. Statistical Analysis

For comparison of the differences between the papain group and vehicle group on both days 3 and 7, we performed the Kruskal–Wallis test. Then, we applied Scheffé’s method for multiple comparisons upon detecting significant differences between different groups. The data have been presented as mean ± standard error (SE), and statistical significance was determined if the *p*-value < 0.05. Furthermore, we performed Spearman’s correlation test to assess the correlation between two variables. Significant or highly significant values were indicated if *p*-value was <0.05, or <0.01, respectively.

## Data Availability

The data presented in this study are available within the article text and figures.

## Supplementary Materials

The following are available online at https://www.mdpi.com/article/10.3390/ijms22136878/s1.

## Abbreviations

BALF	Bronchoalveolar lavage fluid
FALCs	Fat-associated lymphoid clusters
ILCs	Innate lymphoid cells
ILC2s	ILCs group2
Lin	Lineage
MFT	Mediastinal fat tissue
MFALCs	Mediastinal fat-associated lymphoid clusters
NHC	Natural helper cells
d	Days
PBS	Phosphate buffer saline
PAS	Hx-Periodic Acid Schiff-hematoxylin
AAALAC	Association for Assessment and Accreditation of Laboratory Animal Care

## References

[1] Kim H.Y., De Kruyff R.H., Umetsu D.T. (2010). The many paths to asthma: Phenotype shaped by innate and adaptive immunity. Nat. Immunol..

[2] Lambrecht B.N., Hammad H. (2015). The immunology of asthma. Nat. Immunol..

[3] Asher M.I., Montefort S., Björkstén B., Lai C.K., Strachan D.P., Weiland S.K., Williams H. (2006). Worldwide time trends in the prevalence of symptoms of asthma, allergic rhinoconjunctivitis, and eczema in childhood: ISAAC Phases One and Three repeat multicountry cross-sectional surveys. Lancet.

[4] Thomson N.C., Chaudhuri R., Livingston E. (2004). Asthma and cigarette smoking. Eur Respir J..

[5] Milne J., Brand S. (1975). Occupational asthma after inhalation of dust of the proteolytic enzyme, papain. Occup. Environ. Med..

[6] Goeminne P., Adams E., Deschepper K., Valcke Y., Nemery B. (2013). Papain-Induced Asthma: A Man with Dyspnea from Dawn Till Dust. Acta Clinica Belgica.

[7] Halim T., Krauß R.H., Sun A.C., Takei F. (2012). Lung Natural Helper Cells Are a Critical Source of Th2 Cell-Type Cytokines in Protease Allergen-Induced Airway Inflammation. Immunity.

[8] Fukuda K., Matsuzaki H., Hiraishi Y., Mikami Y., Makita K., Nagase T. (2019). Development of papain-induced asthma-COPD overlap mice model. Eur Respir J..

[9] Nials A.T., Uddin S. (2008). Mouse models of allergic asthma: Acute and chronic allergen challenge. Dis. Model. Mech..

[10] Holgate S.T. (2009). Pathogenesis of Asthma. Allergy Allergic Dis..

[11] Barnes P.J. (2008). Immunology of asthma and chronic obstructive pulmonary disease. Nat. Rev. Immunol..

[12] Kay A. (1998). Role of T Cells in Asthma. Mucosal T Cells.

[13] Drake L.Y., Iijima K., Hara K., Kobayashi T., Kephart G.M., Kita H. (2015). B Cells Play Key Roles in Th2-Type Airway Immune Responses in Mice Exposed to Natural Airborne Allergens. PLoS ONE.

[14] Oboki K., Ohno T., Kajiwara N., Arae K., Morita H., Ishii A., Nambu A., Abe T., Kiyonari H., Matsumoto K. (2010). IL-33 is a crucial amplifier of innate rather than acquired immunity. Proc. Natl. Acad. Sci. USA.

[15] Alisjahbana A., Mohammad I., Gao Y., Evren E., Ringqvist E., Willinger T. (2020). Human macrophages and innate lymphoid cells: Tissue-resident innate immunity in humanized mice. Biochem. Pharmacol..

[16] Mills C.D., Kincaid K., Alt J.M., Heilman M.J., Hill A.M. (2000). M-1/M-2 macrophages and the Th1/Th2 paradigm. J. Immunol..

[17] Hirayama D., Iida T., Nakase H. (2017). The Phagocytic Function of Macrophage-Enforcing Innate Immunity and Tissue Homeo-stasis. Int. J. Mol. Sci..

[18] Halim T., Steer C.A., Mathä L., Gold M.J., Martinez-Gonzalez I., McNagny K., McKenzie A.N., Takei F. (2014). Group 2 Innate Lymphoid Cells Are Critical for the Initiation of Adaptive T Helper 2 Cell-Mediated Allergic Lung Inflammation. Immunity.

[19] Moro K., Yamada T., Tanabe M., Takeuchi T., Ikawa T., Kawamoto H., Furusawa J., Ohtani M., Fujii H., Koyasu S. (2010). Innate production of T(H)2 cytokines by adipose tissue-associated c-Kit (+) Sca-1(+) lymphoid cells. Nature.

[20] Elewa Y.H.A., Ichii O., Otsuka S., Hashimoto Y., Kon Y. (2014). Characterization of mouse mediastinal fat-associated lymphoid clusters. Cell Tissue Res..

[21] Elewa Y.H.A., Ichii O., Kon Y. (2015). Comparative analysis of mediastinal fat-associated lymphoid cluster development and lung cellular infiltration in murine autoimmune disease models and the corresponding normal control strains. Immunology.

[22] Elewa Y.H.A., Ichii O., Takada K., Nakamura T., Masum M.A., Kon Y. (2018). Histopathological Correlations between Mediastinal Fat-Associated Lymphoid Clusters and the Development of Lung Inflammation and Fibrosis following Bleomycin Admin-istration in Mice. Front. Immunol..

[23] Zaffini R., Gotte G., Menegazzi M. (2018). Asthma and poly (ADP-ribose) polymerase inhibition: A new therapeutic approach. Drug Des. Dev. Ther..

[24] Johnson J., Wiley R.E., Fattouh R., Swirski F., Gajewska B.U., Coyle A.J., Gutierrez-Ramos J.-C., Ellis R., Inman M.D., Jordana M. (2004). Continuous Exposure to House Dust Mite Elicits Chronic Airway Inflammation and Structural Remodeling. Am. J. Respir. Crit. Care Med..

[25] Fernandez-Rodriguez S., Ford W., Broadley K., Kidd E. (2008). Establishing the phenotype in novel acute and chronic murine models of allergic asthma. Int. Immunopharmacol..

[26] Bousquet J., Jeffery P.K., Busse W.W., Johnson M., Vignola A.M. (2000). Asthma. From bronchoconstriction to airways inflamma-tion and remodeling. Am. J. Respir. Crit. Care Med..

[27] Fish J.E., Peters S.P. (1999). Airway remodeling and persistent airway obstruction in asthma. J. Allergy Clin. Immunol..

[28] Bénézech C., Luu N.T., Walker J.A., Kruglov A.A., Loo Y., Nakamura K., Zhang Y., Nayar S., Jones L.H., Flo-res-Langarica A. (2015). Inflammation induced formation of fat-associated lymphoid clusters. Nat. Immunol..

[29] Elewa Y.H.A., Ichii O., Kon Y. (2017). Sex-related differences in autoimmune-induced lung lesions in MRL/MpJ-*fas*^lpr^ mice are mediated by the development of mediastinal fat-associated lymphoid clusters. Autoimmunity.

[30] Boonyarattanasoonthorn T., Elewa Y.H.A., Tag-El-Din-Hassan H.T., Morimatsu M., Agui T. (2019). Profiling of cellular immune responses to Mycoplasma pulmonis infection in C57BL/6 and DBA/2 mice. Infect. Genet. Evol..

[31] Elewa Y.H., Ichii O., Nakamura T., Kon Y. (2021). Pathological Alternations of Mediastinal Fat-Associated Lymphoid Cluster and Lung in a Streptozotocin-Induced Diabetic Mouse Model. Microsc. Microanal..

