# Supplementary material for: Possible Crosstalk of the Immune Cells within the Lung and Mediastinal Fat-Associated Lymphoid Clusters in the Acute Inflammatory Lung Asthma-Like Mouse Model
Source: Int J Mol Sci. 2021 Jun 26;22(13):6878. doi: 10.3390/ijms22136878 (PMC8268175; doi:10.3390/ijms22136878)
Supplement: Supplementary file 1 [file ijms-22-06878-s001.zip › ijms-1240389-SI.pdf]

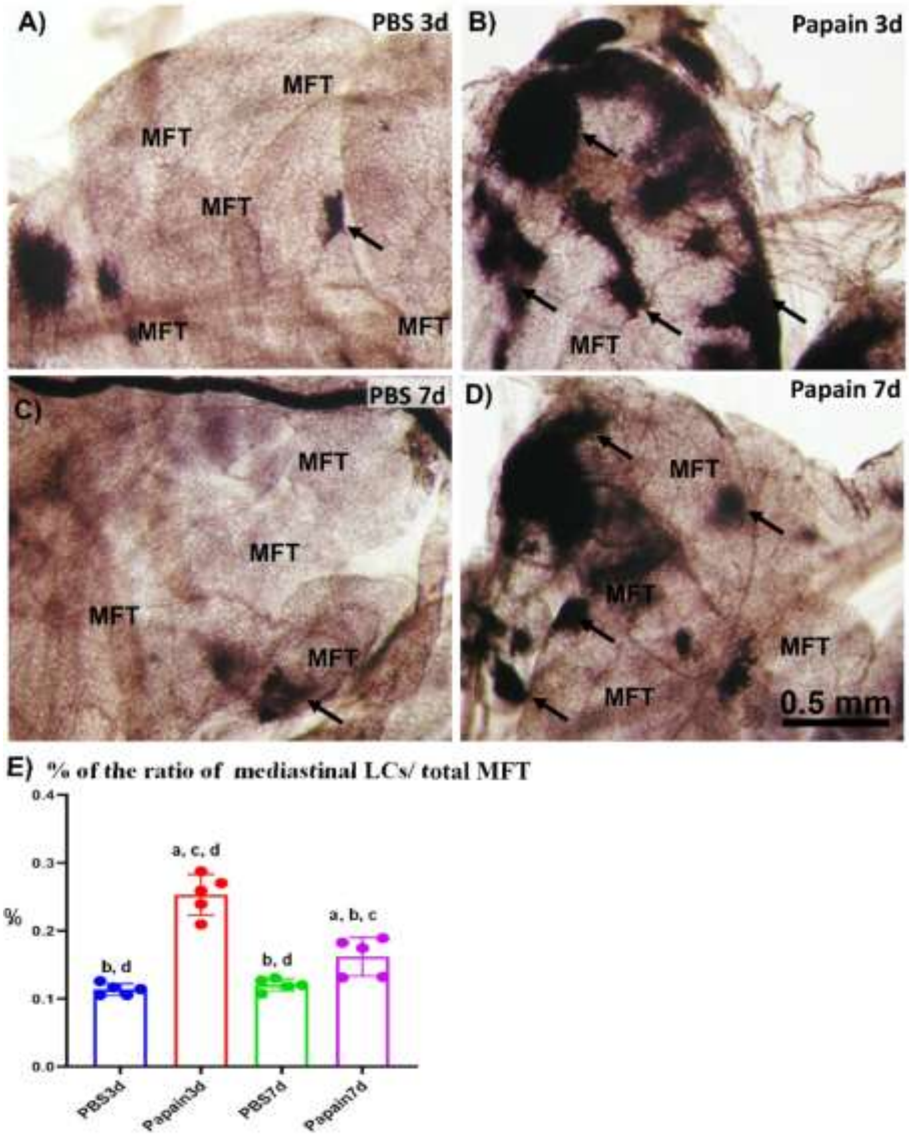

Figure S1. Morphological features and morphometrical analysis of the degree of mediastinal fat-associated lymphoid clusters' (MFALCs') development among papain group (PG) and phosphate buffer saline (PBS) groups (VG) at 3 and 7d. (**A**–**D**) Stereomicrographs of whole-mount hematoxylin-stained mediastinal fat tissue of the PBS groups at 3d (**A**) and 7d (**C**), and papain group at 3d (**B**) and 7d (**D**). Notice LCs (arrows) associating with the mediastinal fat tissue (MFT). (**E**) Graph showing the percentage of the ratio of LCs/total MFT from PG and VG at indicated time points. The letters a, b, c, d: Significant differences between PBS group at 3d (a), papain group at 3d (b), PBS group at 7d (c), and papain group at 7d (d), analyzed by the Kruskal–Wallis test, followed by the Scheffé's method. ($P < 0.05$); n = 5/experimental group. Values are shown as the mean ± SE.

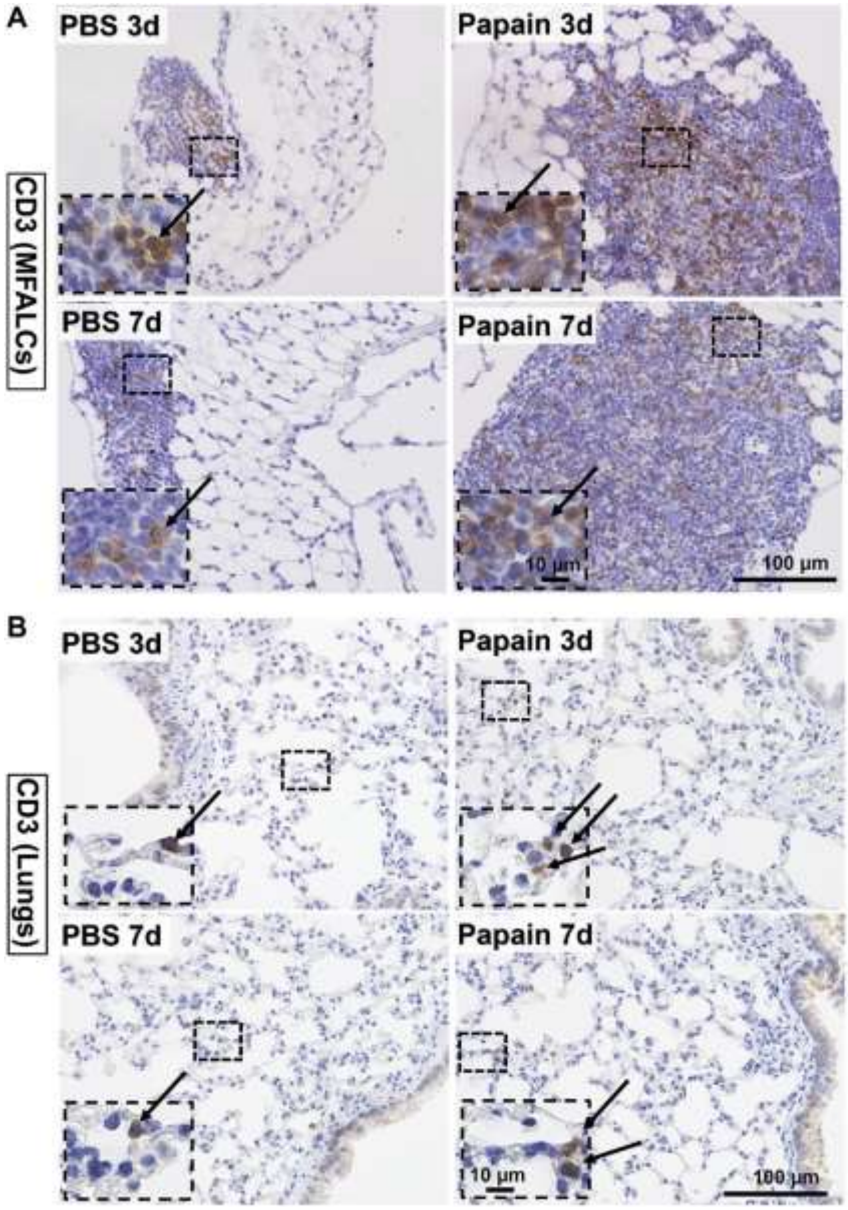

Figure S2. Analysis of the CD3⁺ T-lymphocyte populations in the mediastinal fat-associated lymphoid clusters (MFALCs) and lung of papain group and phosphate buffer saline (PBS) groups at 3 and 7d. (**A**) Immunohistochemical-staining of CD3⁺ T-lymphocytes in mediastinal fat tissue (MFT) (**A**), and lung tissue (**B**) sections of the papain group and vehicle group at indicated time points. Notice CD3⁺ T-lymphocytes (arrows).

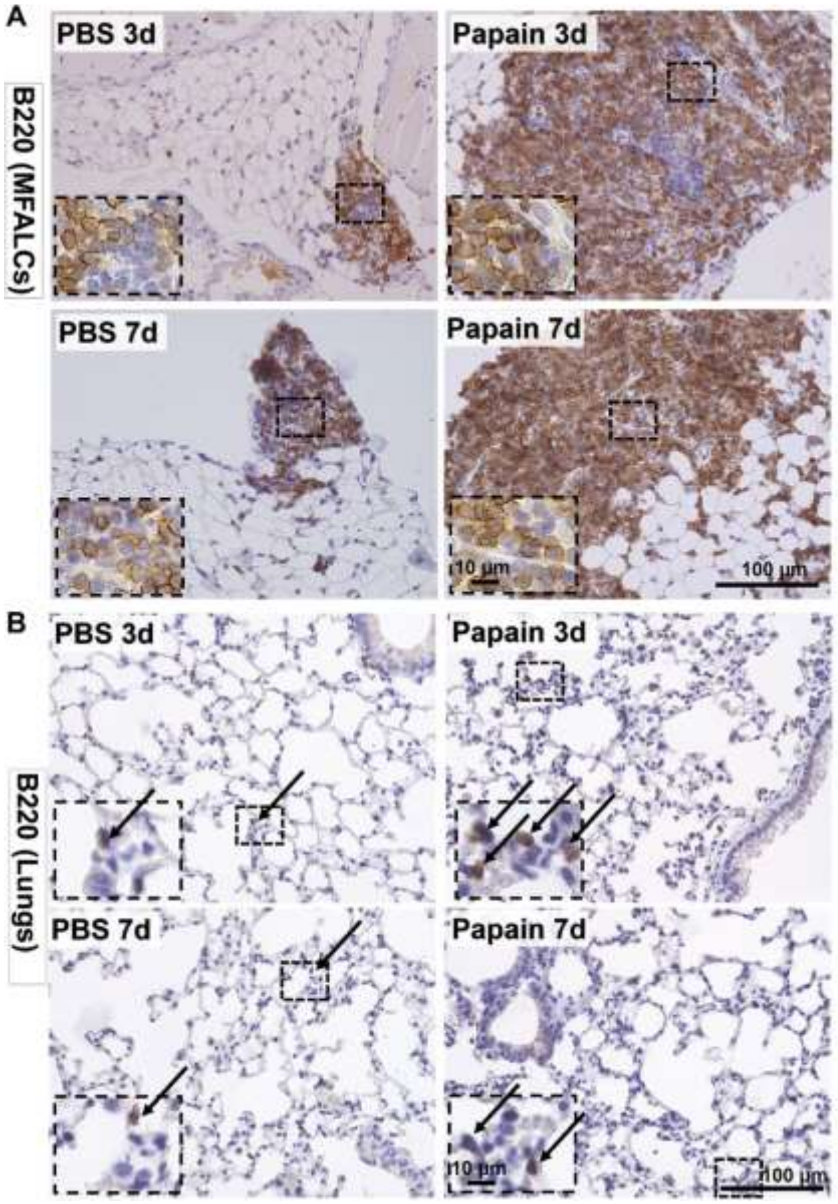

Figure S3. Analysis of the B220$^+$ B-lymphocyte populations in the mediastinal fat-associated lymphoid clusters (MFALCs) and lung of papain group and phosphate buffer saline (PBS) groups at 3 and 7d. (**A**) Immunohistochemical-staining of B220$^+$ B-lymphocytes in mediastinal fat tissue (MFT) (**A**), and lung tissue (**B**) sections of the papain group and vehicle group at indicated time points. Notice B220$^+$ B-lymphocytes (arrows).

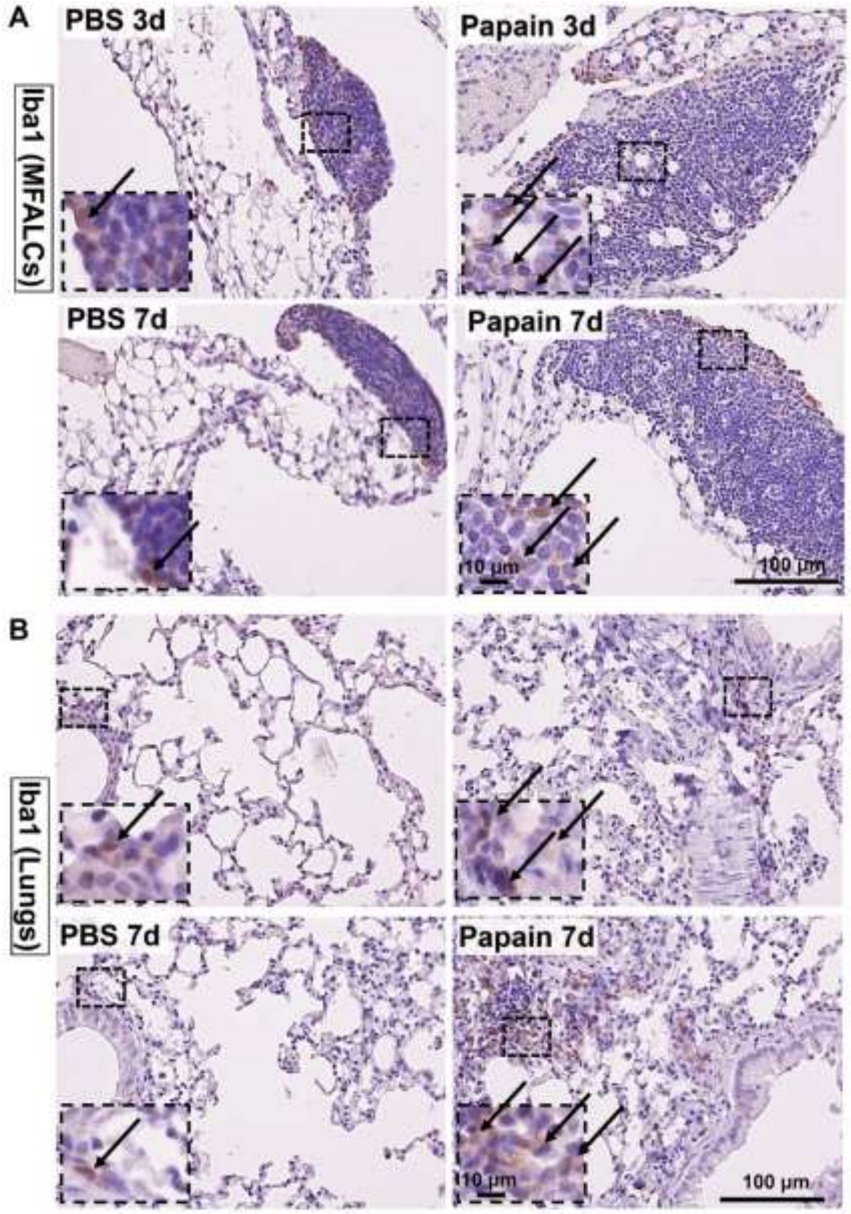

Figure S4. Analysis of the Iba1⁺ macrophages in the mediastinal fat-associated lymphoid clusters (MFALCs) and lung of papain group and phosphate buffer saline (PBS) groups at 3 and 7d. (**A**) Immunohistochemical-staining of Iba1⁺ macrophages in mediastinal fat tissue (MFT) (**A**), and lung tissue (**B**) sections of the papain group and vehicle group at indicated time points. Notice Iba1⁺ macrophages (arrows).

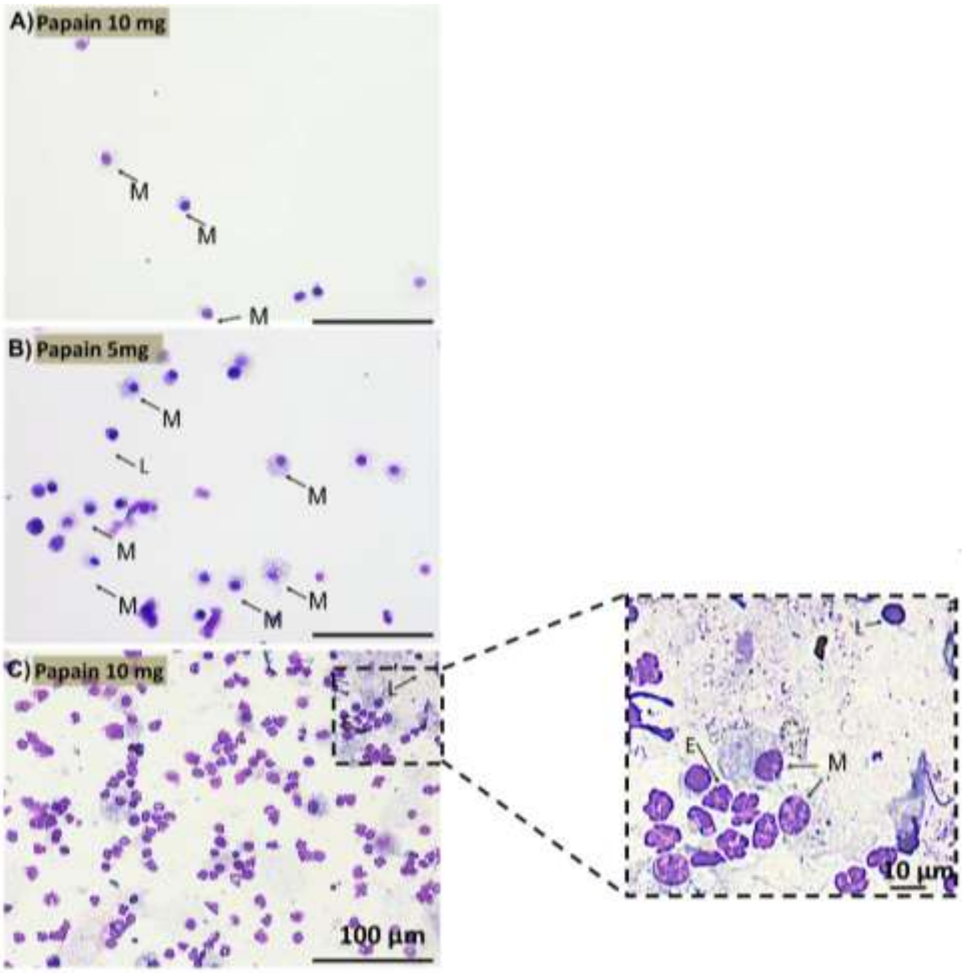

Figure S5. Cytological analysis of the bronchoalveolar lavage fluid (BALF) of phosphate buffer saline (PBS-administered (vehicle)) group and papain groups at different doses (5 and 10 mg). (**A**–**C**) Diff-Quick-stained bronchoalveolar smear at 3d of vehicle group (**A**), papain group administered at 5mg/kg body weight (**B**) and 10mg/kg body weight (**C**). Numerous inflammatory cells (including lymphocytes "L"; macrophages "M"; eosinophils "E", and neutrophils "N") were observed in the papain group (10mg/kg body weight) at 3d (**C**) than other groups.
